# Optimizing the cancer research landscape for the benefit of patients and society: A strategic perspective of the German Cancer Research Center (DKFZ) and its partnerships with university medical centers

**DOI:** 10.20892/j.issn.2095-3941.2022.0586

**Published:** 2022-12-05

**Authors:** Michael Baumann

**Affiliations:** 1 German Cancer Research Center (DKFZ), Heidelberg 69120, Germany

This issue of Cancer Biology & Medicine, a premium Chinese international scientific journal in this field, focuses on cooperation between Chinese and German cancer research, particularly between the Tianjin Medical University Cancer Institute & Hospital and the German Cancer Research Center (DKFZ) with its networking partners. This editorial provides a brief overview on DKFZ’s research strategy with a focus on how a national integrated cancer research and care ecosystem is evolving that benefits patients and society.

## Medical need and general strategy to fight cancer through research

Although considerable progress has been made over the past few decades, cancer continues to be a major and growing health challenge worldwide. In Germany with its population of 83 million, 498,000 people are newly diagnosed with cancer every year^1^ (reference year 2018, excluding non-melanoma skin cancer), and malignant diseases are the leading cause of years of life lost (YLL) in this country (accounting for 35% of YLL)^2^. Cancer incidence in Germany is expected to grow by 20% within the next decade. For Europe, current estimates suggest an increase in the annual incidence of cancer, from 4.4 million in 2020 to 5.3 million in 2040 (+21%)^3^, and globally from 19 million people today to about 30 million in 2040 (+50%). Growing and aging societies, unhealthy lifestyles, and environmental factors are the causes of the increasing cancer burden. There are enormous disparities in incidence and treatment outcome between countries and, albeit to a lesser extent, within nations. Even in the most advanced health systems, including Germany, the 5-year cancer survival rate is only about 65%. 4.65 million people in Germany are living with or after a cancer diagnosis (2017)^4^ and are at risk of developing progressive disease, recurrence, or secondary cancers^1^. Many of them suffer from treatment-related morbidity, sequelae of treatment, or struggle with a reduced quality of life and disadvantages in their professional lives. These alarming global and national trends pose enormous medical, societal and economic challenges that require increased investment in cancer research and care.

**Figure 1:**
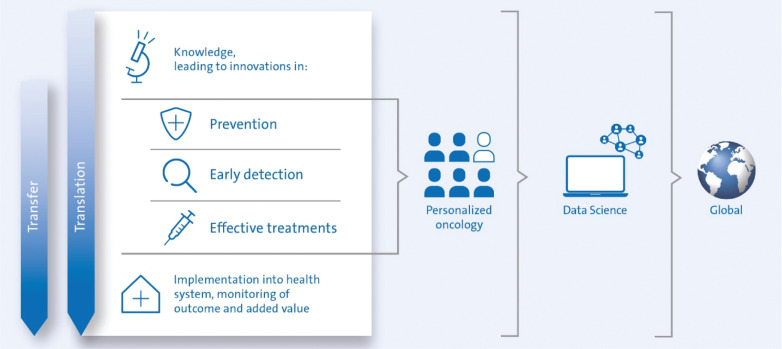
Strategies to decrease cancer incidence and death through research (adapted from Reference 5).

Major challenges need to be tackled to reduce the burden of cancer and improve cancer treatment. Fundamental discoveries in basic research are pushing the frontiers of knowledge towards a better understanding of cancer biology or for the basis of new technologies, diagnostics or medications. To achieve substantial clinical progress, these new discoveries must be applied through translational and clinical research in three main areas (**Figure 1**)^5^:

- Cancer prevention: up to 40% of cancer cases can be avoided by minimizing or eliminating known risk factors^6,7^ (i.e. primary prevention). New risk factors and pathogenetic mechanisms leading to cancer need to be uncovered in order to further develop strategies counteracting the rising cancer incidence. Preventive measures offered to patients with or after cancer can reduce the risk of recurrence or unwarranted sequelae (i.e. tertiary prevention).
- Early detection: new diagnostic methods and strategies need to be developed to recognize tumors at earlier, and thus potentially curable stages (i.e. secondary prevention). Such strategies can be biomarker-based (liquid-, tissue- or breath biomarkers) or image-based; often both modalities are necessary to obtain a lab-test warning sign, which is followed-up by imaging.
- Novel and improved clinical treatments: increasingly efficient and precise diagnostics and treatments are expected to further improve cancer cure rates. In addition, new treatments have the potential to transform advanced cancers from an acutely lethal disease into a chronic, manageable disease. Novel strategies of personalized precision oncology in an integrated multidisciplinary and participatory clinical setting promise higher anti-tumor efficacy, fewer side effects and an overall better quality of life for patients.

Cancer is a uniqueIy heterogenous disease. Significant biological differences exist not only between tumor entities but equally between different patients, between different lesions of the same patient, within each individual lesion in a single patient and at different time points during disease progression and therapy. Therefore, all three broad anticancer research strategies increasingly require personalized approaches, are highly dependent on progress in data-science, and benefit from global cooperation.

Finally, once new anti-cancer strategies have been developed in clinical or basic prevention research, society needs concepts for their efficient implementation in the health system to make these innovations widely available. In addition, the benefits of such innovations need to be constantly monitored and scrutinized through health economic and outcome research.

## Research at the DKFZ

The mission of the DKFZ is to make a significant contribution to decrease new cancer cases and cancer deaths – through transformative research, education of the next generation of leaders, and the transfer of results into the health system, society, and innovative products. DKFZ is primarily funded by the German Federal Ministry of Education and Research (BMBF) and the federal states in which the main center or its branches are located. DKFZ is a member of the Helmholtz Association, the largest German research organization^8^.

**Figure 2:**
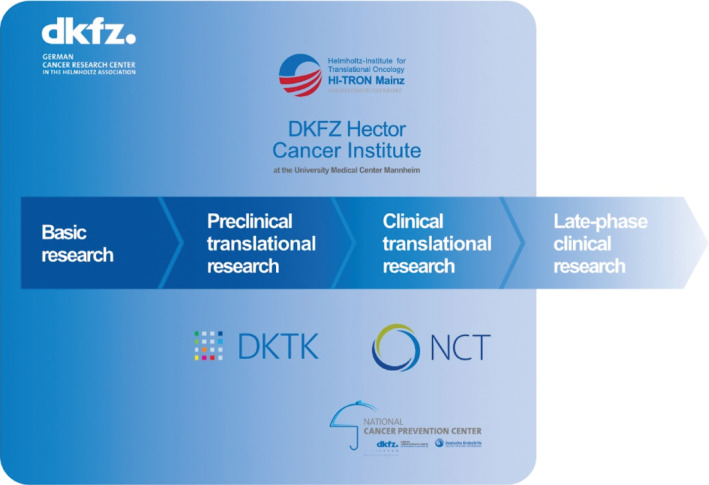
Positioning of the DKFZ and its networks with University Medical Centers within the translational continuum (adapted from References 9 and 10).

Currently, more than 3,000 staff members, including more than 1,300 scientists, doctoral researchers and clinician scientists, are performing basic, preclinical and clinical translational research at the DKFZ. Additionally, more than 1,500 associated or guest scientists have access to research facilities at the center in Heidelberg or at branch locations. Research covers the entire translational continuum from discovery of fundamental causes of cancer to the development of disruptive, preventive, diagnostic and therapeutic approaches, and to innovative clinical studies/trials (**Figure 2**)^9,10^. The DKFZ has particular strength in areas such as stem cell research; cancer genomics; cancer prevention; tumor immunology, inflammation and infection; imaging, radiooncology and image-guided therapies. As transformative innovations often originate at the interface between different areas, DKFZ promotes an increasing number of temporary or long-term ‘horizontal’ cross topic programs including single-cell approaches, digital oncology including AI-driven approaches, or disease-specific areas such as neuro-oncology or pediatric oncology. The DKFZ Cancer Research Academy hosts the International PhD Program, one of Europe’s largest graduate programs in the biomedical field, the International Postdoc Program and the International Clinician Scientist Program as well as specialized further activities, to educate, train and mentor the next generation of leaders for tomorrow’s needs. Several advisories help the DKFZ to formulate and continuously adapt its strategy, including scientific advisory boards, a patient advisory board, and a council assembling members from industry, politics, the public sphere and communication experts.

## Strengthening the translational research continuum by joint structures and networks

**Figure 3:**
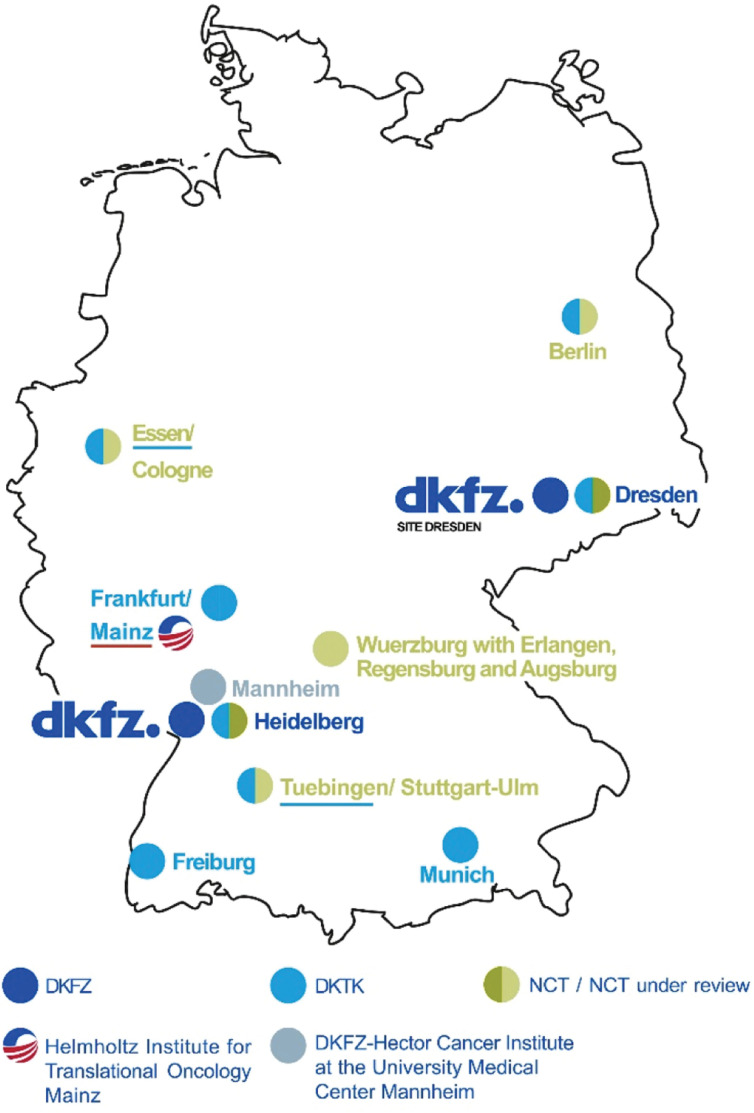
The DKFZ and its institutional branches (adapted from References 9 and 10).

Discovery and basic cancer research at universities and academic research centers in Germany have been at the international forefront for decades. For example, the discovery of Human Papillomaviruses (HPV) as a cause of cervical cancer by 2008 Nobel laureate Harald zur Hausen in the 1970s and early 80s laid the ground for later efforts outside of Germany in vaccine development and vaccination trials that have since provided clear evidence for the prevention of this disease. Another example is the theranostic agent ^177^Lu-PSMA-617 for the treatment of metastatic castrate-resistant prostate cancer, which was invented and brought into first-in-human application at the DKFZ and the University Hospital Heidelberg. However, most early phase clinical trials testing innovations of laboratories in Germany as well as the vast majority of registration trials, e.g. the VISION III study for FDA approval of ^177^Lu-PSMA-617, are presently performed outside Germany^11^. Overall, the targeted translation of cutting-edge results from basic research and preclinical pipelines into early clinical trials and particularly transfer into innovative products is still a major challenge in Germany. To tackle this challenge and work towards a complete and integrated translational research and transfer continuum, the DKFZ has established institutional branches for translational and clinical cancer research together with leading university medical centers (UMCs) in Germany in recent years. These include bilateral platforms such as the Helmholtz Institute for Translational Oncology Mainz (HI-TRON Mainz), and the DKFZ-Hector Cancer Institute in Mannheim as well as multi-site networks: The German Cancer Consortium (DKTK) and the National Center for Tumor Diseases (NCT) (**Figure 3**)^9,10^. The emerging cooperative network is increasingly comprehensive, although there are still open areas, particularly in northern Germany. With the National Decade against Cancer (2019–2028), the BMBF has launched a unique initiative with the aim to significantly advance cancer research in Germany, to create strong and sustainable structures necessary for future challenges and to ensure that all people in Germany have access to high-quality oncological care and to innovations in cancer research – regardless of where they live.

### German Cancer Consortium (DKTK)

The DKTK was initiated by the BMBF in 2012 to provide significant contributions to bridging the gap (‘valley of death’) between basic cancer research and clinical cancer research/clinical prevention research using state-of-the-art translational and reverse translational approaches. For this, DKFZ, as the consortium’s core center, together with local university hospitals and further partners, has established translation hubs at eight sites (Berlin, Dresden, Essen/Duesseldorf, Frankfurt/Mainz, Freiburg, Heidelberg, Munich, and Tuebingen) all of which are in cancer research and care. The aim is to further develop personalized oncology approaches and to improve early detection, diagnosis and therapy of cancer. Since its inception, DKTK has implemented long-term sustained programs, innovative research platforms, and established new full professorships and junior research groups at the eight partner sites. The DKTK network trains tomorrow’s leaders in translational cancer research, with a particular focus on clinician scientists and medical scientists. In addition, DKTK has established an intramural Joint Funding Program to launch annual competitive calls for site-overarching research activities. DKTK has evolved into a powerful innovation hub that actively integrates its bridging role in the translational research continuum, and provides a platform bringing together the best cancer researchers in the country. An impressive example of success is the very prominent role played by DKTK scientists in the development of new molecular classifications for brain tumors and pediatric cancers, which have been adopted by the WHO and are now globally used in clinical practice^12,13^.

### National Center for Tumor Diseases (NCT)

The next step within the translational research chain, namely innovative clinical translational cancer research, is still a critical bottleneck in Germany. To address this bottleneck, the NCT was founded to integrate patient-centered and translational-driven clinical cancer research with top-notch multidisciplinary cancer care. The NCT has the potential to overcome current shortcomings by creating powerful and interconnected clinical translational trial infrastructures and expertise.

In 2003, the DKFZ established a first NCT site in Heidelberg in collaboration with the University Hospital Heidelberg and the *Deutsche Krebshilfe* (foundation). The NCT integrates a number of central activities “under one roof” in a dedicated building, including the most important elements of multidisciplinary outpatient care and innovative clinical trial activities, particularly in the field of personalized oncology. In 2014, the BMBF decided to fund the existing NCT site in Heidelberg and a second NCT site in Dresden. These NCT sites have proved to be successful models for fostering innovative clinical cancer research, including clinical trials fueled by own preclinical pipelines. As a central element of the *National Decade Against Cancer*, the federal government therefore decided to expand the NCT by up to four new sites to create greater critical mass and comprehensiveness as well as to facilitate improved access to innovative clinical trials for patients nationwide. The UMCs of (i) Berlin (NCT Berlin), (ii) Cologne and Essen (NCT West), (iii) Tuebingen-Stuttgart with Ulm (NCT SouthWest), and (iv) Wuerzburg with Erlangen, Regensburg and Augsburg (NCT WERA) were selected by international peer review as candidates for the expanded NCT. They are currently working with the two existing NCTs and DKFZ on a joint strategy and implementation plan which will undergo peer-review before start of the program.

### National Cancer Prevention Center (NCPC)

The NCPC, which is currently being established in a strategic partnership between the DKFZ and the *Deutsche Krebshilfe*, will pilot an innovative concept of personalized cancer prevention. The concept is modeled on the experience of the Comprehensive Cancer Centers, which have shown that integration of research and multidisciplinary clinical services significantly promote translation, innovation and quality of care as well as of research. Located in a new dedicated building which will be constructed at the DKFZ Heidelberg campus, the NCPC will combine biological research on mechanisms of oncogenesis and biomarkers with translational, clinical and outcome prevention and early detection research. Consultation offices and laboratories for citizens and patients seeking advice or wishing to participate in interventional prevention trials will be next door to research laboratories. Prototypic evidence-based workflows, a digital prevention unit, database and biomaterial archives, and comprehensive education programs for the next generation of prevention experts will feed into large-scale nationwide outreach initiatives.

## International cooperation

The DKFZ’s strategy for internationalization aims to create synergies by teaming up with strategically selected cancer and cancer research centers of excellence around the world that ideally complement the DKFZ cancer research program. In addition to a wide spectrum or multitude of international collaborative efforts among individual scientists and within international research networks, DKFZ supports several major international partnerships at a formalized institutional level. Examples are the long-lasting bi-national DKFZ-MOST (Ministry of Science and Technology) program between scientists from the DKFZ and academic institutions in Israel, the collaboration between DKFZ and INSERM with a joint research unit in Heidelberg, the sister institution relationship with the MD Anderson Cancer Center in Houston, or joint initiatives with the US National Cancer Institute NCI, the International Agency for Research and Cancer (IARC), Cancer Research UK, Tianjin Medical University Cancer Institute & Hospital, King Hussein Cancer Center in Jordan, University and Health Ministry of Namibia, or the emerging Athens Cancer Center. DKFZ is also a key partner in leading European consortia like Cancer Core Europe and Cancer Prevention Europe. Examples for joint international efforts in education and training are a joint research school with the Weizmann Institute of Science in Israel or the International Clinician and Medical Scientist exchange program with the Princess Margaret Cancer Center in Toronto.

## Perspectives

DKFZ, together with its partners at the UMCs and further institutions, works towards a coherent and high-performing cancer research landscape in Germany that spans the entire translational continuum and can effectively support excellent cancer researchers to generate impact for cancer patients and society. This long-term national investment into an integrated cancer research and care environment also serves as a powerful basis for close international partnerships to effectively fight cancer on a global scale.
